# Equivalency of four research-grade movement sensors to assess movement behaviors and its implications for population surveillance

**DOI:** 10.1038/s41598-022-09469-2

**Published:** 2022-04-01

**Authors:** Jairo H. Migueles, Pablo Molina-Garcia, Lucia V. Torres-Lopez, Cristina Cadenas-Sanchez, Alex V. Rowlands, Ulrich W. Ebner-Priemer, Elena D. Koch, Andreas Reif, Francisco B. Ortega

**Affiliations:** 1 PROFITH “PROmoting FITness and Health Through Physical Activity” Research Group, Sport and Health University Research Institute (iMUDS), Department of Physical Education and Sports, Faculty of Sport Sciences, University of Granada, Granada, Spain; 2 Department of Biosciences and Nutrition, Karolinska Institutet, Huddinge, Sweden; 3 Institute for Innovation & Sustainable Development in Food Chain (IS-FOOD), Department of Health Sciences, Public University of Navarra, IdiSNA, Navarra Institute for Health Research, Pamplona, Spain; 4 Diabetes Research Centre, Leicester General Hospital, University of Leicester, Gwendolen Road, Leicester, LE5 4PW UK; 5 NIHR Leicester Biomedical Research Centre, University Hospitals of Leicester NHS Trust and the University of Leicester, Leicester, LE5 4PW UK; 6 Alliance for Research in Exercise, Nutrition and Activity (ARENA), Sansom Institute for Health Research, Division of Health Sciences, University of South Australia, Adelaide, SA 5001 Australia; 7 Department of Psychiatry and Psychotherapy, Central Institute of Mental Health, Medical Faculty Mannheim/Heidelberg University, Mannheim, Germany; 8 Mental mHealth Lab, Institute of Sports and Sports Science, Karlsruhe Institute of Technology (KIT), Karlsruhe, Germany; 9 Department of Psychiatry, Psychosomatic Medicine and Psychotherapy, University Hospital Frankfurt, Goethe University, Frankfurt, Germany; 10 Faculty of Sport and Health Sciences, University of Jyväskylä, Jyväskylä, Finland

**Keywords:** Risk factors, Population screening

## Abstract

the benefits of physical activity (PA) and sleep for health, accurate and objective population-based surveillance is important. Monitor-based surveillance has potential, but the main challenge is the need for replicable outcomes from different monitors. This study investigated the agreement of movement behavior outcomes assessed with four research-grade activity monitors (i.e., Movisens Move4, ActiGraph GT3X+, GENEActiv, and Axivity AX3) in adults. Twenty-three participants wore four monitors on the non-dominant wrist simultaneously for seven days. Open-source software (GGIR) was used to estimate the daily time in sedentary, light, moderate-to-vigorous PA (MVPA), and sleep (movement behaviors). The prevalence of participants meeting the PA and sleep recommendations were calculated from each monitor’s data. Outcomes were deemed equivalent between monitors if the absolute standardized difference and its 95% confidence intervals (CI_95%_) fell within ± 0.2 standard deviations (SD) of the mean of the differences. The participants were mostly men (n = 14, 61%) and aged 36 (SD = 14) years. Pairwise confusion matrices showed that 83–87% of the daily time was equally classified into the movement categories by the different pairs of monitors. The between-monitor difference in MVPA ranged from 1 (CI_95%_: − 6, 7) to 8 (CI_95%_: 1, 15) min/day. Most of the PA and sleep metrics could be considered equivalent. The prevalence of participants meeting the PA and the sleep guidelines was 100% consistent across monitors (22 and 5 participants out of the 23, respectively). Our findings indicate that the various research-grade activity monitors investigated show high inter-instrument reliability with respect to sedentary, PA and sleep-related estimates when their raw data are processed in an identical manner. These findings may have important implications for advancement towards monitor-based PA and sleep surveillance systems.

## Introduction

In the light of the unquestionable benefits of physical activity (PA) on humans’ health^1,2^, and the globally estimated physical inactivity levels^3,4^, it is of utmost importance to establish population-based surveillance systems for regular PA assessment and reporting^5,6^. Self-reports are the most common method of surveillance as they are inexpensive, unobtrusive, and adaptable to different contexts. However, most of the self-reports used in surveillance systems present considerable measurement errors and desirability bias^7–9^. Instead, as suggested by the World Health Organization (WHO), activity monitors can theoretically strengthen PA surveillance with more accurate device-based measures^10^. Self-reports assess a person’s perception and recall of their movement behavior, while devices enable assessment of movement behavior through the continuous recording of the accelerations produced by a certain body part (e.g., arm, hip, thigh). Likewise, devices could potentially expand surveillance to other related variables, such as sedentary behavior and sleep. Yet there also exist challenges relative to the use of wearable technology for surveillance^5,10^.

Challenges related to consumer-marketed activity monitors are data ownership, population representativeness, the short lifespan of devices, and the use of proprietary non-replicable algorithms^5^. The deployment of research-grade monitors (i.e., devices specifically developed for research purposes; they usually provide no feedback to participants and allow to download the raw accelerations) by public health and surveillance systems could overcome most of these limitations. However, the replicability of the measures obtained from different devices would be a major concern^11,12^, since it is unfeasible that the same monitor would be used by all surveillance systems worldwide. For example, Japan has repeatedly used pedometers (Yamasa Co, Ltd, Tokyo, Japan) for PA surveillance^1313^, Canada has collected PA measures with the Actical (Philips Respironics, Oregon, USA)^14^, the USA used ActiGraph devices (ActiGraph, Pensacola FL, USA) in the National Health and Nutrition Examination Survey (NHANES)^15,16^, or the UK opted for the ActiGraph in the Health Survey for England^17^.

Until 2010, most accelerometers provided only proprietary count data limiting comparability between devices, being the most widely used, the “counts” from ActiGraph^18^. Modern research-grade devices have enough battery life and storage capacity to provide the raw data collected, theoretically facilitating the generation of replicable outcomes from across brands. Open-source software, developed in the field, has the capacity to process these raw data using identical methods, irrespective of monitor brand, e.g., GGIR^19^. Whether the resulting movement behavior estimates (e.g., time in PA intensities, sedentary time, and sleep-related outcomes) are compatible across devices when the raw data are processed using consistent methods is still an open question. Some factors that could influence the comparability across brands are the monitor size, the sensor specifications (e.g., sampling frequency, dynamic range), and the body attachment site where the monitor is placed. Previous research have tested the comparability of the same monitor across different body attachment sites, with promising findings for data harmonization across studies^20^.

Therefore, this study aimed to investigate the agreement of the daily time spent in various PA intensities, sedentary time, and sleep-related outcomes assessed with four different research-grade activity monitors (i.e., Movisens, ActiGraph, GENEActiv, and Axivity) in young adults. Based on previous research^11,21,22^, we expect that differences in the movement behaviors estimates are small to negligible when the raw data from different monitors are processed similarly. The selected monitors are within the most-frequently used in research^18^, and/or have been used in previous large cohorts^23–27^. The wrist was selected as some of the largest cohorts are collecting data from wrist-worn accelerometers in their recent data collections^23,26,27^. How different acceleration metrics compare across body sites using the same monitor has been previously reported^20^. Additionally, the wrist provide a range of accelerations higher than other body sites, which makes the comparison between devices richer as it expands to higher movement intensities.

## Results

The 23 participants included were 61% male (n = 14), aged 36 (SD = 14) years, and with a mean BMI of 26 (SD = 5.5) kg/m^2^ (Table 1). Mean and SD of daily time accumulated in sleep, sedentary time, light, and MVPA are reported in Table 2.

**Table 1:** Descriptive characteristics of participants.

Characteristics	All (n = 23)	Men (n = 14)	Women (n = 9)
Age (years)	36.04 (14.18)	33.21 (14.67)	40.44 (12.93)
Weight (kg)	75.62 (15.93)	76.99 (14.96)	73.5 (18.06)
Height (cm)	171.08 (10.59)	176.2 (9.82)	163.11 (5.86)
BMI (kg/m^2^)	25.88 (5.46)	24.82 (4.89)	27.52 (6.17)
**Weight status, nr. (%)**			
Underweight	2 (9%)	1 (7%)	1 (11%)
Normal weight	10 (43%)	8 (57%)	2 (22%)
Overweight	7 (30%)	3 (22%)	4 (45%)
Obesity	4 (18%)	2 (14%)	2 (22%)

Data presented as mean (SD) or frequency (percentage) as appropriate.

*BMI* body mass index, *SD* standard deviation.

**Table 2:** Mean and SD for sedentary time, light PA, MVPA, and sleep, as determined with the different activity monitors (i.e., Movisens, ActiGraph, GENEActiv, and Axivity).

	Movisens	ActiGraph	GENEActiv	Axivity
Sleep period time (min/day)	401 ± 66	399 ± 69	410 ± 69	396 ± 68
Sedentary (min/day)	651 ± 115	659 ± 118	636 ± 111	660 ± 12
Light (min/day)	184 ± 64	179 ± 64	184 ± 64	183 ± 58
MVPA (min/day)	101 ± 52	99 ± 49	106 ± 52	98 ± 48

*PA* physical activity, *SD* standard deviation.

**Figure 1:**
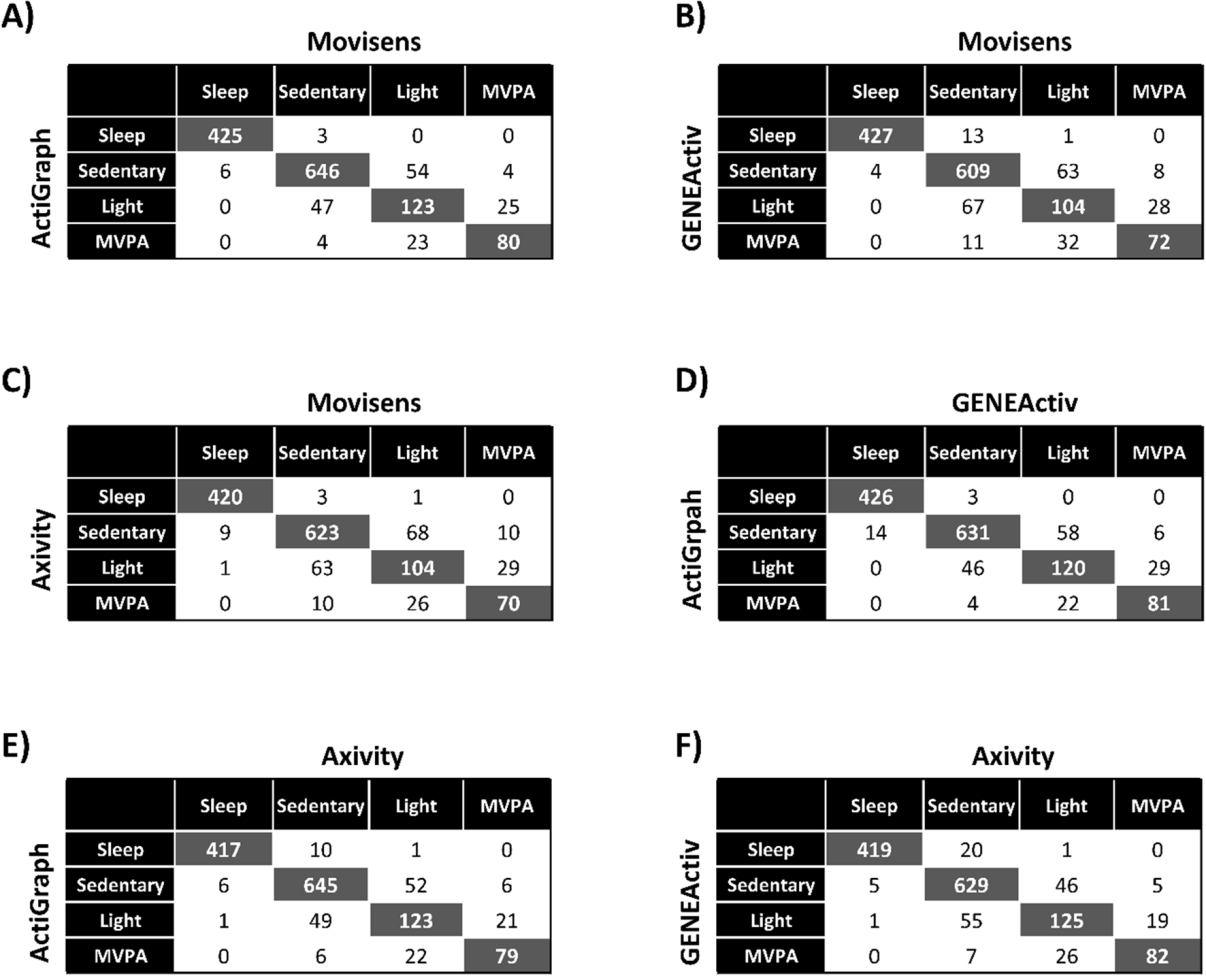
Pairwise confusion matrices for the sleep, sedentary time, light PA and MVPA expressed in min/day as determined by the different monitors. *MVPA* moderate-to-vigorous physical activity, *PA* physical activity.

Confusion matrices for the classification of behaviors (i.e., sleep, sedentary time, light, and MVPA) between each pair of monitors are shown in Fig. 1. For each metric, most of the daily time is equally classified between each pair of monitors, i.e., epochs equally classified (gray cells, Fig. 1)/epochs differently classified (white cells, Fig. 1) * 100 (Movisens vs. ActiGraph: 87% of the day; Movisens vs. GENEActiv: 83% of the day; Movisens vs. Axivity: 84% of the day; ActiGraph vs. GENEActiv: 86% of the day; ActiGraph vs. Axivity: 87% of the day; GENEActiv vs. Axivity: 86% of the day).

Table 3 shows the sensitivity and specificity values for the classification of the behaviors obtained with each monitor compared to the Movisens (i.e., reference). The sensitivity was substantial for light PA classified by the ActiGraph (i.e., 0.61), and moderate for the GENEActiv (i.e., 0.52) and the Axivity (i.e., 0.52) compared to the Movisens. In regards to MVPA, the sensitivity values were substantial for the ActiGraph (i.e., 0.73), GENEActiv (i.e., 0.66), and Axivity (i.e., 0.64) compared to the Movisens. The rest of sensitivity and specificity values using the Movisens as reference were almost perfect for all the metrics (i.e., ≥ 0.87). Similar findings were observed after alternating the referent monitor (i.e., moderate to substantial sensitivity for light and MVPA between pairs of monitors; supplementary material, Tables S1, S2 and S3).

**Table 3:** Agreement between Movisens-defined and the other monitors (i.e., ActiGraph, GENEActiv and Axivity) in the definition of sleep, sedentary time, light PA, and MVPA.

	Movisens			
	Sleep	Sedentary	Light PA	MVPA
**Movisens**				
min/d	431	700	199	110
**ActiGraph**				
Sensitivity	0.99	0.92	0.61	0.73
Specificity	1.00	0.91	0.94	0.98
**GENEActiv**				
Sensitivity	0.99	0.87	0.52	0.66
Specificity	0.99	0.90	0.92	0.97
**Axivity**				
Sensitivity	0.98	0.89	0.52	0.64
Specificity	1.00	0.88	0.92	0.97

*MVPA* moderate-to-vigorous physical activity, *PA* physical activity.

**Figure 2:**
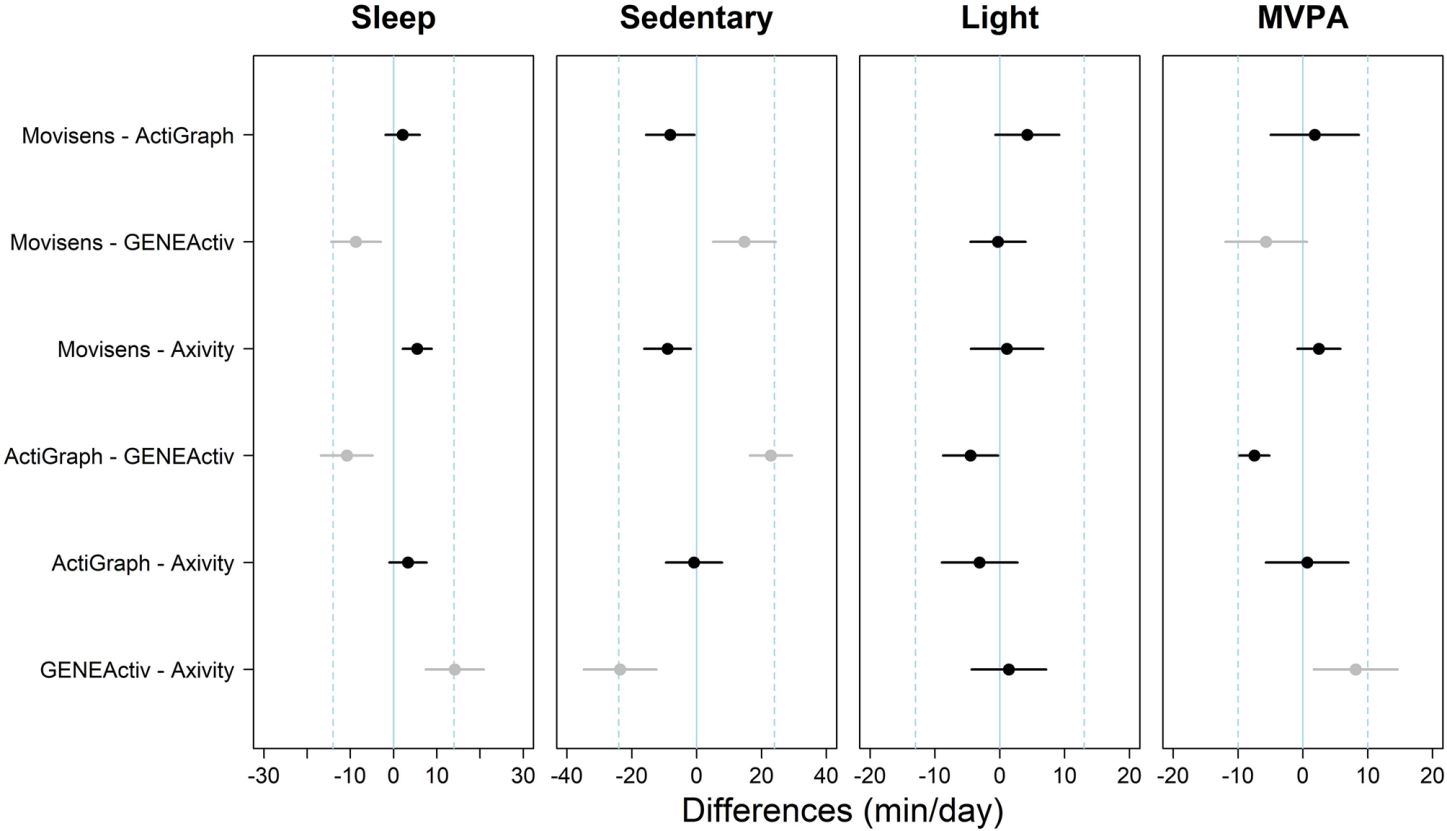
Equivalence between pairs of monitors for sedentary time, light, moderate, and vigorous PA. Points represent the difference in min/day between a pair of monitors (as determined in the vertical axis) for every PA metric. Error bars represent the CI_95%_ of the difference. Perfect equivalence = 0 (solid line); proposed equivalence zone (± 0.2 SDs of the differences) represented by dashed lines. Black markers indicate that the CI_95%_ are within the equivalence zone, and grey markers indicate that they go beyond the equivalence zone. *CI*_*95%*_ 95% confidence intervals, *PA* physical activity, *SD* standard deviation.

The equivalency between pairs of monitors for the metrics investigated is shown in Fig. 2. Regarding sleep and sedentary time time, the Movisens, the ActiGraph and the Axivity were deemed equivalent, yet these monitors were not equivalent to the GENEActiv. For light PA, all the monitors were equivalent as the CI_95%_ of the differences fell within the equivalency band. In regards to MVPA, the GENEActiv was not equivalent to the Movisens and the Axivity, while the rest of pairwise comparisons resulted in equivalent values. All monitors agreed on the prevalence of our sample which met the WHO PA guidelines (i.e., 22 out of the 23 participants reached the recommendation) and the national sleep foundation guidelines on sleep time (i.e., 5 out of the 23 participants).

Supplemental Figures S1 to S6 show high correlations between pairs of monitors for all the metrics studied (all r’s ≥ 0.947). Bland–Altman plots with the mean difference and 95% limits of agreement between pairs of monitors for the PA and sleep metrics can be found in the supplementary material (Figs. S7 to S12). No trends or heteroscedasticity were observed in any of the Bland–Altman plots performed.

## Discussion

The main findings from this study indicate that the prevalence of our participants meeting the WHO PA guidelines and the sleep foundation guidelines was exactly the same when determined by the different monitors (i.e., Movisens Move 4, ActiGraph GT3X+, GENEActiv, and Axivity AX3). However, this finding should be considered with caution since we have a rather small and non-representative sample. The monitors agreed in the classification of individual behaviors over 84% of the daily time for all pairings of monitors. We observed high sensitivity and specific values for the classification of sleep and sedentary time for all pairings; yet moderate-to-substantial sensitivities for the classification of light and MVPA and high specificities for these behaviors. Furthermore, the various research-grade activity monitors investigated provided equivalent estimations of the daily time spent in sleep, sedentary time, light, and MVPA. Some exceptions were observed in the comparisons including the GENEActiv monitor, likely due to the mounting of the device in this study. These findings stand as long as the raw data of these monitors are processed in an identical manner, which was allowed in this study by the open-source software GGIR^19^. Altogether, these findings may have important implications to advance towards monitor-based PA and sleep surveillance systems.

Some previous studies have investigated the agreement between different research-grade activity monitors using consistent raw data processing methods^11,12,28^. Rowlands et al. investigated the equivalency of the ActiGraph, the GENEActiv, and the Axivity for the accelerations recorded (sampling frequency: 100 Hz, dynamic range: ± 8 g) in the non-dominant wrist in different lab-based activities. They found that time spent sedentary and in light PA could be considered equivalent for all monitors, but time in MVPA only for the GENEActiv and Axivity^11^. The fact that the GENEActiv and the Axivity were taped together to the same wristband, while the ActiGraph was independently attached and their location was not counterbalanced, may partially account for this finding. Standardized mounting of devices might be important for PA surveillance. In this study, we found similar compatibility between monitors attached to different wristbands (e.g., Movisens vs ActiGraph or ActiGraph vs Axivity) compared with monitors in the same wrist band (e.g., Movisens vs Axivity). The fact that the GENEActiv was slightly less compatible with the other devices in this study might be because it was attached laterally to the wrist. This position might have produced some extra noise in the acceleration signal and might have been more disturbing for participants, which might affect the compatibility with the other devices. Furthermore, the ecology of lab-based activities to infer conclusions over free-living behaviors is questionable. In another study by Rowlands et al., they compared the accelerations recorded during a two-day free-living assessment between the ActiGraph and the GENEActiv^12^. Among the metrics, they included the daily time in MVPA and sleep and between-brand differences were negligible (i.e., 4 min/day for MVPA and 1 min/day for sleep). Plekhanova et al. found equivalent values for sleep between the ActiGraph, the GENEActiv and the Axivity collecting data at a sampling frequency of 100 Hz and a dynamic range of ± 8 g^29^.

Our study expands the previous studies by Rowlands et al.^11,12^ by including a seven-day free-living assessment, focusing on time in different movement behaviors frequently used in public health research and epidemiology^30^, including another monitor (the Movisens), and counterbalancing the order of the wristbands across participants. In this regard, we found the Axivity and the ActiGraph were equivalent for all the metrics, while the GENEActiv was not equivalent with these monitors for sleep, sedentary and MVPA, which is contrary to the Rowlands et al. findings^11^. However, our findings agree in that the time spent in the estimated PA and sleep-related behaviors were consistent across devices as Rowlands et al. found for sedentary time^11,12^, MVPA and sleep time^12^. Otherwise, Crowley et al. compared the ActiGraph and the Axivity worn on the thigh for the identification of certain behaviors (i.e., sitting, standing, walking, running, stair climbing, cycling, or stepping) in free-living conditions^28^. Overall, they found small differences between the ActiGraph and the Axivity (e.g., 3 min/day walking, resulting in 323 steps/day difference). Our findings agree with these in the small differences across devices when the raw data are processed in a consistent manner. Although caution is advised because the different body attachment site complicates the comparability between our findings and those by Crowley et al.^28^ as the range of accelerations recorded from the wrist is expected to be higher than the thigh, which could exert higher between-brand differences.

The WHO called for the development and testing of digital technologies to strengthen the population PA surveillance^10^. Some concerns have been raised regarding the use of consumer-marketed activity monitors, such as the influence of real-time feedback on screens, the data ownership, population representativeness, the short lifespan of device, and proprietary non-replicable algorithms^5^. The deployment of research-grade devices by public health and surveillance systems might overcome these limitations. Data ownership and population representativeness would be overcome as the surveillance agency would own the data and design the procedures for the data collection to ensure adequate population representativeness. Regarding the lifespan, research-grade devices stay in the market much longer than consumer-marketed devices. For example, the ActiGraph GT3X + has been (and is still) available since 2010, while consumer-marketed devices lifespan is usually 1–2 years. Furthermore, research-grade monitor manufacturers try to ensure data comparability across different generations of their monitors (e.g., ActiGraph GT3X + and GT9X Link). Therefore, the replicability of the algorithms applied to the raw data would be the major concern for monitor-based PA surveillance, which enhances the value of open-source algorithms.

Although proprietary algorithms and data replicability have also been a major concern with former research-grade activity monitors^31^, important advances to enhance the data comparability have occurred since the monitors allow access to the raw data collected. This study, together with previous evidence^11,12,28^, demonstrate that it is possible to obtain similar estimations of PA and sleep-related behaviors from different monitors as long as the raw data are processed consistently. The comparability of the raw data collected mainly relies on the components integrated in the different devices and their physical characteristics (e.g., weight, size). Although some of the manufacturers provide detailed information of their components, some others hide such information. We encourage manufacturers to be transparent about the components and mounting of their devices, so that researchers can consider the similarities and differences across devices when designing the studies. It is noticeable that we found the same proportion of our study sample meeting the WHO guidelines on aerobic PA (i.e., 150 min per week of MVPA) and the sleep foundation guidelines (i.e., 7 to 8 h of sleep per day). However, caution is advised in the interpretation of this finding since we have a convenient, small, and non-representative sample. Future studies with larger and representative samples should corroborate this promising finding. What seems unquestionable from the evidence available is that there is potential to use different brands of research-grade accelerometers to obtain comparable estimates of movement behaviors and the proportion of people meeting PA and sleep guidelines, as long as the activity monitors allow for the storage of the raw data to be processed in a consistent manner with open-source and replicable algorithms.

The findings of this study should be interpreted under the consideration of its limitations: (i) the sample size analyzed is rather small (n = 23); (ii) it is a convenience sample which does not represent the population, although BMI was quite heterogeneous; (iii) the mounting of devices was not usual as we fitted four devices in the same wrist (e.g., the effect of proximality of the wristband was not tested; we encourage future studies to investigate the device mounting and its relevance for the movement behaviors assessment); (iv) the accelerometers settings (e.g., sampling frequency, dynamic range) were not identical across monitors, although we used the closest settings allowed by each brand; (v) differences in the accelerometer sensor components could not be considered since this information is not made publicly available by all the manufacturers included in this study; and (v) we lack a criterion for the measurement of the PA and sleep-related behaviors. A criterion would have provided information on the accuracy of the devices, but it does not affect our main objective, which is to study the agreement across different activity monitors for the estimation of PA and sleep-related behaviors. Otherwise, some strengths of this study are worthy of mention: (i) we compared research-grade activity monitors which are widely used in the PA measurement field^18^, and have been used in large cohorts for surveillance and epidemiological studies; and (ii) we processed the raw data of the monitors using the same protocol and open-source algorithms in the GGIR software, which is also widely used in the field^19^.

In conclusion, our findings indicate that the various research-grade activity monitors investigated (i.e., Movisens Move 4, ActiGraph GT3X + , GENEActiv, and Axivity AX3) have a high agreement (> 80%) for estimations of the daily time spent in sleep, sedentary time, light, and MVPA when their raw data are processed in an identical manner. Importantly, the prevalence of our participants meeting the WHO PA guidelines was identical when determined by the different monitors. These findings may have important implications for advancement towards monitor-based PA and sleep surveillance systems.

## Methods

### Study design and participants

**Figure 3:**
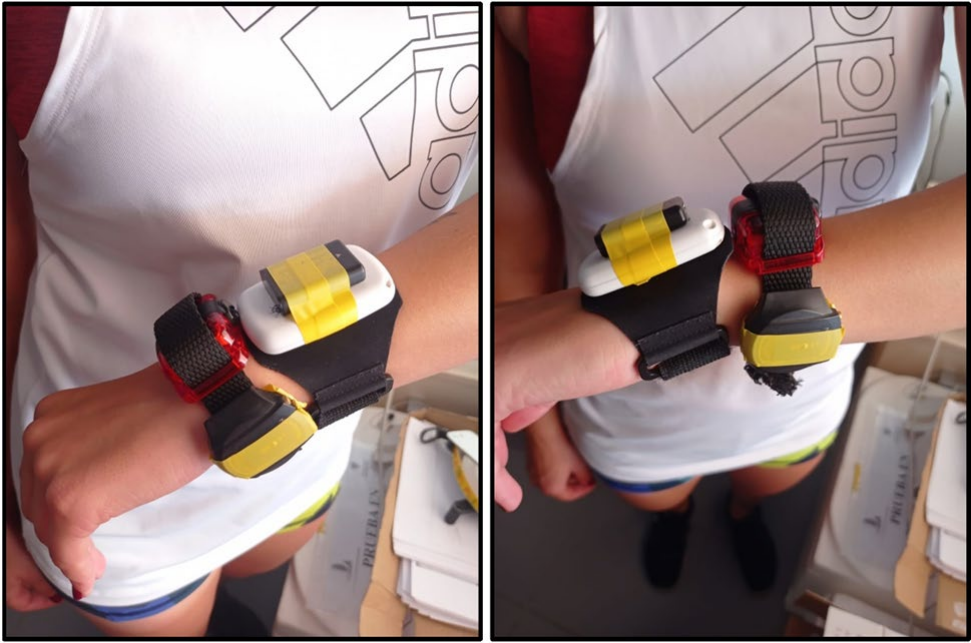
Attachment of the devices in two wristbands. The proximality of the wristbands was counterbalanced across participants.

The data analyzed in this study were collected in Granada (Spain) as a local branch of the EU-funded CoCA project (“**Co**morbid **C**onditions of **A**ttention-deficit/hyperactivity disorder”). CoCA aims to provide new knowledge and tools to prevent adolescent and young adult attention-deficit/hyperactivity disorder from escalating into detrimental comorbidities (https://coca-project.eu/). Among other treatments, the CoCA project tests the effect of a mHealth-deployed exercise program with continuous PA monitoring with the Movisens Move 4 (Movisens GmbH, Karlsruhe, Germany). One of the secondary aims of CoCA was to investigate the convergent validity of this activity monitor against widely-used research-grade monitors in the PA measurement field. As such, we recruited a convenience sample of 25 young adults from the AMPACHICO association (Granada, Spain). We asked them to wear four activity monitors on the non-dominant wrist for seven days (i.e., the Movisens Move 4, the ActiGraph GT3X+, the GENEActiv [ActivInsights Ltd., Cambridgeshire, UK], and the Axivity AX3). The activity monitors were placed on participants’ non-dominant wrist attached to two wristbands. The Movisens and the Axivity were attached to one wristband, and the ActiGraph and the GENEActiv to the other (Fig. 3). The order of the wristbands regarding the proximality to the body was counterbalanced. Two participants were excluded for not wearing the Movisens/Axivity wristband (n = 1), or for not wearing any monitor during the sleeping periods (n = 1). Thus, 23 participants were included in these analyses. All participants provided written informed consent, and this study was approved by the Ethics Committee on Human Research (CEIH) of the University of Granada. This study was conducted according to the Declaration of Helsinki.

### Activity monitors

The Movisens Move 4 contains a triaxial accelerometer which captures accelerations within a dynamic range of ± 16 *G*’s (where 1 *G* represents the gravitational acceleration, i.e., ~ 9.8 m/s^2^) at a sampling frequency of 64 Hz. The ActiGraph GT3X + is a monitor which includes a triaxial accelerometer with a dynamic range of ± 8 *G*’s. The sampling rate can be set from 30 to 100 Hz in increments of 10 Hz, for this study, a sampling rate of 60 Hz was set. The “idle sleep mode” was disabled for this monitor. The GENEActiv contains a triaxial accelerometer capturing accelerations in a range of ± 8 *G*’s; it was set at a sampling rate of 60 Hz. Lastly, the Axivity AX3 is the smallest of the monitors used in this study, and contains a triaxial accelerometer with a configurable dynamic range, which was set to ± 8 *G*’s for this study. The sampling rate was set at 50 Hz in the Axivity AX3. The sampling rates in the ActiGraph, the GENEActiv, and the Axivity were selected to be as close as their respective software allowed to the sampling frequency of the Movisens (which is fixed to 64 Hz). Likewise, the dynamic range was pre-fixed by manufacturers for all the devices, except for the Axivity, in which we selected 8 *G*’s to approximate most of the rest of devices. We used 10 units of each device for the data collection.

Raw data from the monitors were downloaded in the respective software made available by their manufacturers. This is, the Movisens Move 4 files were downloaded in the SensorManager software (Movisens GmbH, Karlsruhe, Germany) in binary format; the ActiGraph files were downloaded and converted to csv files in the ActiLife software v.6.13.4 (ActiGraph, Pensacola FL, USA); the GENEActiv raw data were downloaded using the GENEActiv PC software (ActivInsights Ltd., Cambridgeshire, UK) as binary files; and the Axivity files were downloaded in the OmGui open-source software (OmGui, Open Movement, Newcastle University, Newcastle upon Tyne, UK) and saved in cwa format.

### Raw data processing

Raw data from all monitors were processed in the open-source R package GGIR^19^. The processing methods were the same for all devices, and they involved: (i) autocalibration of the data according to the local gravity^32^; (ii) calculation of the Euclidean Norm Minus One *G* with negative values rounded to zero (ENMO) over 5-s epochs; (iii) detection of the non-wear time based on the standard deviation (SD) and magnitude of each axis’ raw acceleration^33^; (iv) detection of sustained abnormal high accelerations (i.e., each acceleration recorded close to the dynamic range limits of each monitor, for example accelerations higher than 7.5 g’s for the ActiGraph, GENEActiv and Axivity); (v) classification of waking and sleeping times with an automated algorithm^34,35^; (vi) removal of every epoch classified as non-wear time or abnormal high acceleration by at least one of the monitors. Time in sleep (time from sleep onset to wake up), sedentary (< 35 mg), light (35–99.9 mg), and moderate-to-vigorous (MVPA, > 100 mg) physical activities were considered for analyses^34–37^.

### Sociodemographic data and anthropometrics

As part of the protocol, participants reported their age and sex; and we measured their body weight and height to the nearest 0.1 kg and 0.1 cm using an electronic scale (SECA 861, Hamburg, Germany) and a precision stadiometer (SECA 225, Hamburg, Germany), respectively. Body mass index (BMI) was calculated as kg/m^2^.

### Data analysis

Participants’ descriptive data were reported as mean and SD or frequencies and percentages, as appropriate. All analyses were performed with the reference monitor selected according to this hierarchy: Movisens, ActiGraph, GENEActiv and Axivity. This decision was arbitrary as none of the monitors used can be considered the gold standard. Confusion matrices were built between each pair of monitors with the minutes per day classified in each time-based category (i.e., sleep, sedentary time, light, and MVPA). Then, sensitivity (i.e., true positives) and specificity (i.e., true negatives) values were calculated for each monitor, using the Movisens as the reference. Sensitivity and specificity values were considered slight (0.00–0.20), fair (0.21–0.40), moderate (0.41–0.60), substantial (0.61–0.80), or almost perfect (0.81–1.00) following pre-defined standards^38^. To explore if the referent monitor affected the findings, we alternated the monitor used as reference in this analysis. Next, the equivalence between each pair of monitors was investigated by determining whether the mean difference and 95% confidence intervals (CI_95%_) for each pair of monitors fell within a proposed equivalence zone. To account for the specific variability in each metric investigated, the equivalence zone was defined as ± 0.2 SDs from the mean of the between-monitor differences, as this is the minimum relevant standardized difference considered by the Cohen’s D standards^39^. Likewise, bivariate correlations and Bland–Altman plots were drawn for each pair of monitors to investigate the agreement between the metrics of interest as determined by the different monitors. Finally, the prevalence of participants meeting the WHO PA guidelines (i.e., ≥ 150 min per week of MVPA)^1^ and the national sleep foundation guidelines (i.e., 7 to 8 h of sleep per day)^40^ were determined based on the total time in MVPA per week and total sleep time per day estimated from each monitor.

## Supplementary Information

Supplementary Information.

## References

[1] Bull F (2020). World Health Organization 2020 guidelines on physical activity and sedentary behaviour. Br. J. Sports Med..

[2] Chaput JP (2020). 2020 WHO guidelines on physical activity and sedentary behaviour for children and adolescents aged 5–17 years: Summary of the evidence. Int. J. Behav. Nutr. Phys. Act..

[3] Kohl HW (2012). The pandemic of physical inactivity: Global action for public health. Lancet.

[4] Lee I-M (2012). Effect of physical inactivity on major non-communicable diseases worldwide: An analysis of burden of disease and life expectancy. Lancet.

[5] Troiano RP, Stamatakis E, Bull FC (2020). How can global physical activity surveillance adapt to evolving physical activity guidelines? Needs, challenges and future directions. Br. J. Sports Med..

[6] Organization WH (2004). Global Strategy on Diet, Physical Activity and Health.

[7] Shephard RJ (2003). Limits to the measurement of habitual physical activity by questionnaires. Br. J. Sports Med..

[8] Shaw PA (2018). Epidemiologic analyses with error-prone exposures: Review of current practice and recommendations. Ann. Epidemiol..

[9] Matthews CE (2018). Measurement of active and sedentary behavior in context of large epidemiologic studies. Med. Sci. Sports Exerc..

[10] World Health Organization (2018). Global Action Plan on Physical Activity 2018–2030: More Active People for a Healthier World.

[11] Rowlands AV (2018). Accelerometer-assessed physical activity in epidemiology: Are monitors equivalent?. Med. Sci. Sports Exerc..

[12] Rowlands AV, Yates T, Davies M, Khunti K, Edwardson CL (2016). Raw accelerometer data analysis with GGIR R-package: Does accelerometer brand matter?. Med. Sci. Sports Exerc..

[13] Takamiya T, Inoue S (2019). Trends in step-determined physical activity among Japanese adults from 1995 to 2016. Med. Sci. Sports Exerc..

[14] Clarke J, Colley R, Janssen I, Tremblay MS (2019). Accelerometer-measured moderate-to-vigorous physical activity of Canadian adults, 2007 to 2017. Health Rep..

[15] Troiano RP (2008). Physical activity in the United States measured by accelerometer. Med. Sci. Sports Exerc..

[16] Troiano RP, McClain JJ, Brychta RJ, Chen KY (2014). Evolution of accelerometer methods for physical activity research. Br. J. Sports Med..

[17] Joint Health Surveys Unit. *Health Survey for England 2008: Volume 1. Physical activity and fitness,* **1**, 395 http://content.digital.nhs.uk/catalogue/PUB00430/heal-surv-phys-acti-fitn-eng-2008-rep-v2.pdf (2008).

[18] Wijndaele K (2015). Utilization and harmonization of adult accelerometry data: Review and expert consensus. Med. Sci. Sports Exerc..

[19] Migueles JH, Rowlands AV, Huber F, Sabia SS, van Hees VT (2019). GGIR: A research community-driven open source r package for generating physical activity and sleep outcomes from multi-day raw accelerometer data. J. Meas. Phys. Behav..

[20] Migueles JH (2019). Comparability of accelerometer signal aggregation metrics across placements and dominant wrist cut points for the assessment of physical activity in adults. Sci. Rep..

[21] Brønd JC, Arvidsson D, Brond JC, Arvidsson D (2015). Sampling frequency affects the processing of Actigraph raw acceleration data to activity counts. J. Appl. Physiol..

[22] Shapero K (2016). Cardiovascular risk and disease among masters endurance athletes: Insights from the Boston MASTER (Masters Athletes Survey To Evaluate Risk) initiative. Sport. Med..

[23] Doherty A (2017). Large scale population assessment of physical activity using wrist worn accelerometers: The UK biobank study. PLoS ONE.

[24] Evenson KR (2021). Cohort profile: The Women’s Health Accelerometry Collaboration. BMJ Open.

[25] Marmot MG (1991). Health inequalities among British civil servants: The Whitehall II study. Lancet.

[26] Sabia S (2014). Association between questionnaire-and accelerometer-assessed physical activity: The role of sociodemographic factors. Am. J. Epidemiol..

[27] John D, Tang Q, Albinali F, Intille S (2019). An Open-source monitor-independent movement summary for accelerometer data processing. J. Meas. Phys. Behav..

[28] Crowley P (2019). Comparison of physical behavior estimates from three different thigh-worn accelerometers brands: A proof-of-concept for the Prospective Physical Activity, Sitting, and Sleep consortium (ProPASS). Int. J. Behav. Nutr. Phys. Act..

[29] Plekhanova T (2020). Equivalency of sleep estimates: Comparison of three research-grade accelerometers. J. Meas. Phys. Behav..

[30] Migueles JH (2021). GRANADA consensus on analytical approaches to assess associations with accelerometer-determined physical behaviours (physical activity, sedentary behaviour and sleep) in epidemiological studies. Br. J. Sports Med..

[31] Migueles JH (2017). Accelerometer data collection and processing criteria to assess physical activity and other outcomes: A systematic review and practical considerations. Sports Med..

[32] Van Hees VT (2014). Auto-calibration of accelerometer data for free-living physical activity assessment using local gravity and temperature: An evaluation on four continents. J. Appl. Physiol..

[33] van Hees VT (2013). Separating movement and gravity components in an acceleration signal and implications for the assessment of human daily physical activity. PLoS ONE.

[34] van Hees VT (2018). Estimating sleep parameters using an accelerometer without sleep diary. Sci. Rep..

[35] Van Hees VT (2015). A novel, open access method to assess sleep duration using a wrist-worn accelerometer. PLoS ONE.

[36] Hildebrand M, Van Hees VT, Hansen BH, Ekelund U (2014). Age group comparability of raw accelerometer output from wrist- and hip-worn monitors. Med. Sci. Sports Exerc..

[37] Hildebrand M, Hansen BH, van Hees VT, Ekelund U (2017). Evaluation of raw acceleration sedentary thresholds in children and adults. Scand. J. Med. Sci. Sports.

[38] Landis JR, Koch GG (1977). The measurement of observer agreement for categorical data. Biometrics.

[39] Cohen J (1992). A power primer. Psychol. Bull..

[40] Hirshkowitz M (2015). National sleep foundation’s sleep time duration recommendations: Methodology and results summary. Sleep Health.

