# Supplementary material for: Equivalency of four research-grade movement sensors to assess movement behaviors and its implications for population surveillance
Source: Sci Rep. 2022 Apr 1;12:5525. doi: 10.1038/s41598-022-09469-2 (PMC8975935; doi:10.1038/s41598-022-09469-2)
Supplement: Supplementary file 1 — Supplementary Information. [file 41598_2022_9469_MOESM1_ESM.docx]

**Supplementary material**

**Table S1.** Agreement between ActiGraph-defined and the other monitors (i.e., GENEActiv, Axivity, and Movisens) in the definition of sleep, sedentary time, light, and MVPA.

|  |  |  | **ActiGraph** | | | |
| --- | --- | --- | --- | --- | --- | --- |
|  |  |  | **Sleep** | **Sedentary** | **Light PA** | **MVPA** |
| **ActiGraph** | min/d |  | 428 | 709 | 195 | 108 |
| **ActiGraph** | Sensitivity |  | 0.99 | 0.89 | 0.61 | 0.75 |
|  | Specificity |  | 0.99 | 0.93 | 0.94 | 0.97 |
| **GENEActiv** | Sensitivity |  | 0.97 | 0.91 | 0.63 | 0.74 |
|  | Specificity |  | 0.99 | 0.91 | 0.94 | 0.98 |
| **Axivity** | Sensitivity |  | 0.99 | 0.91 | 0.63 | 0.75 |
|  | Specificity |  | 0.99 | 0.93 | 0.94 | 0.98 |

MVPA: moderate-to-vigorous physical activity; PA: physical activity

**Table S2.** Agreement between GENEActiv-defined and the other monitors (i.e., ActiGraph, Axivity, and Movisens) in the definition of sleep, sedentary time, light PA, and MVPA.

|  |  |  | **GENEActiv** | | | |
| --- | --- | --- | --- | --- | --- | --- |
|  |  |  | **Sleep** | **Sedentary** | **Light PA** | **MVPA** |
| **GENEActiv** | min/d |  | 440 | 684 | 200 | 116 |
| **ActiGraph** | Sensitivity |  | 0.97 | 0.92 | 0.60 | 0.70 |
|  | Specificity |  | 1.00 | 0.90 | 0.94 | 0.98 |
| **GENEActiv** | Sensitivity |  | 0.95 | 0.92 | 0.62 | 0.71 |
|  | Specificity |  | 0.99 | 0.89 | 0.94 | 0.98 |
| **Axivity** | Sensitivity |  | 0.97 | 0.89 | 0.52 | 0.63 |
|  | Specificity |  | 1.00 | 0.88 | 0.92 | 0.97 |

MVPA: moderate-to-vigorous physical activity; PA: physical activity.

**Table S3.** Agreement between Axivity-defined and the other monitors (i.e., ActiGraph, GENEActiv, and Movisens) in the definition of sleep, sedentary time, light PA, and MVPA.

|  |  |  | **Axivity** | | | |
| --- | --- | --- | --- | --- | --- | --- |
|  |  |  | **Sleep** | **Sedentary** | **Light PA** | **MVPA** |
| **Axivity** | min/d |  | 425 | 711 | 198 | 107 |
| **ActiGraph** | Sensitivity |  | 0.98 | 0.91 | 0.62 | 0.74 |
|  | Specificity |  | 0.99 | 0.91 | 0.94 | 0.98 |
| **GENEActiv** | Sensitivity |  | 0.99 | 0.88 | 0.63 | 0.77 |
|  | Specificity |  | 0.98 | 0.92 | 0.94 | 0.98 |
| **Axivity** | Sensitivity |  | 0.99 | 0.88 | 0.53 | 0.66 |
|  | Specificity |  | 0.99 | 0.89 | 0.92 | 0.97 |

MVPA: moderate-to-vigorous physical activity; PA: physical activity.

**
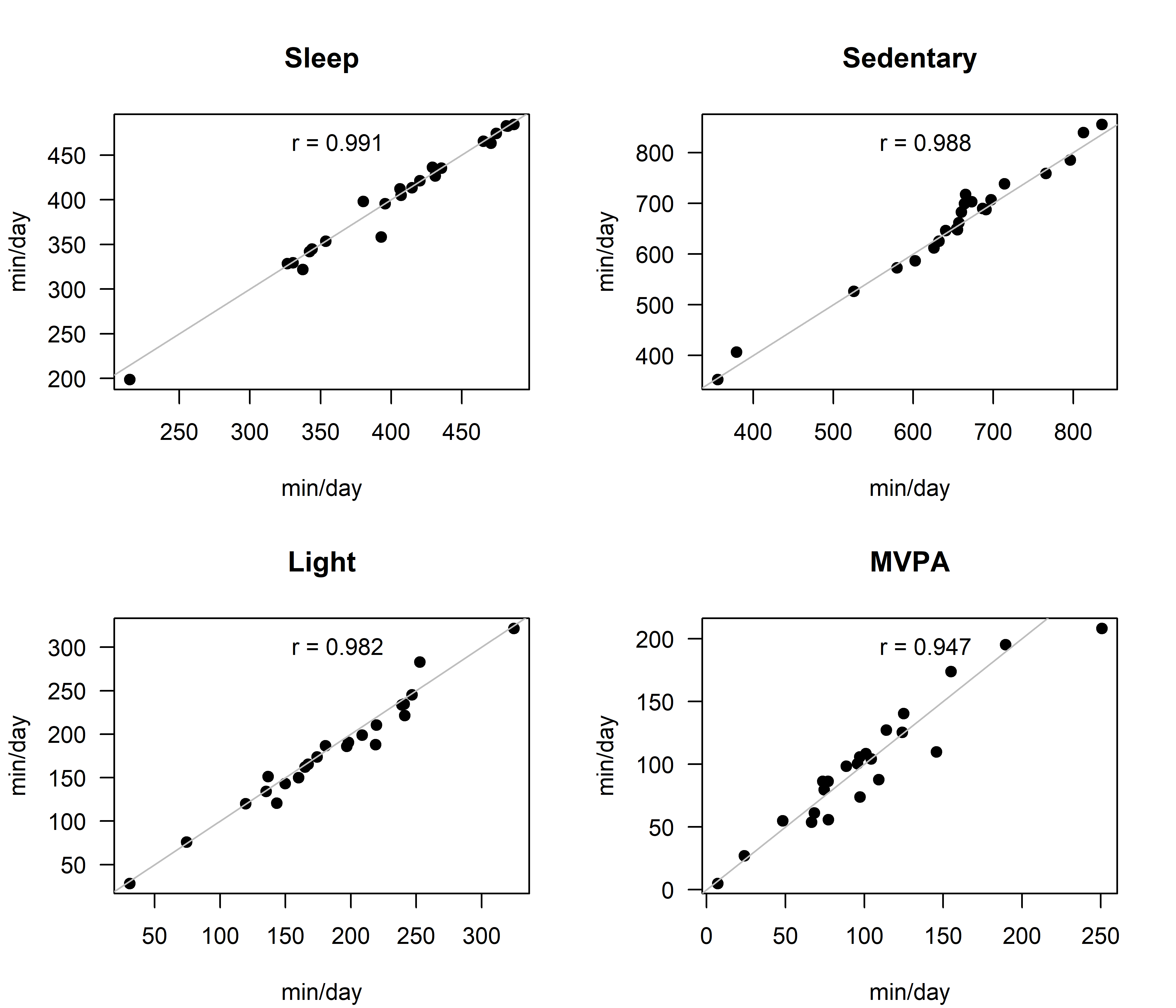
**

**Figure S1.** Scatter plots for the correlation between **the Movisens and the ActiGraph** in the definition of sleep, sedentary time, light PA, and MVPA. The grey line represents the perfect correlation (i.e., r = 1).

MVPA: moderate-to-vigorous physical activity; PA: physical activity.

**
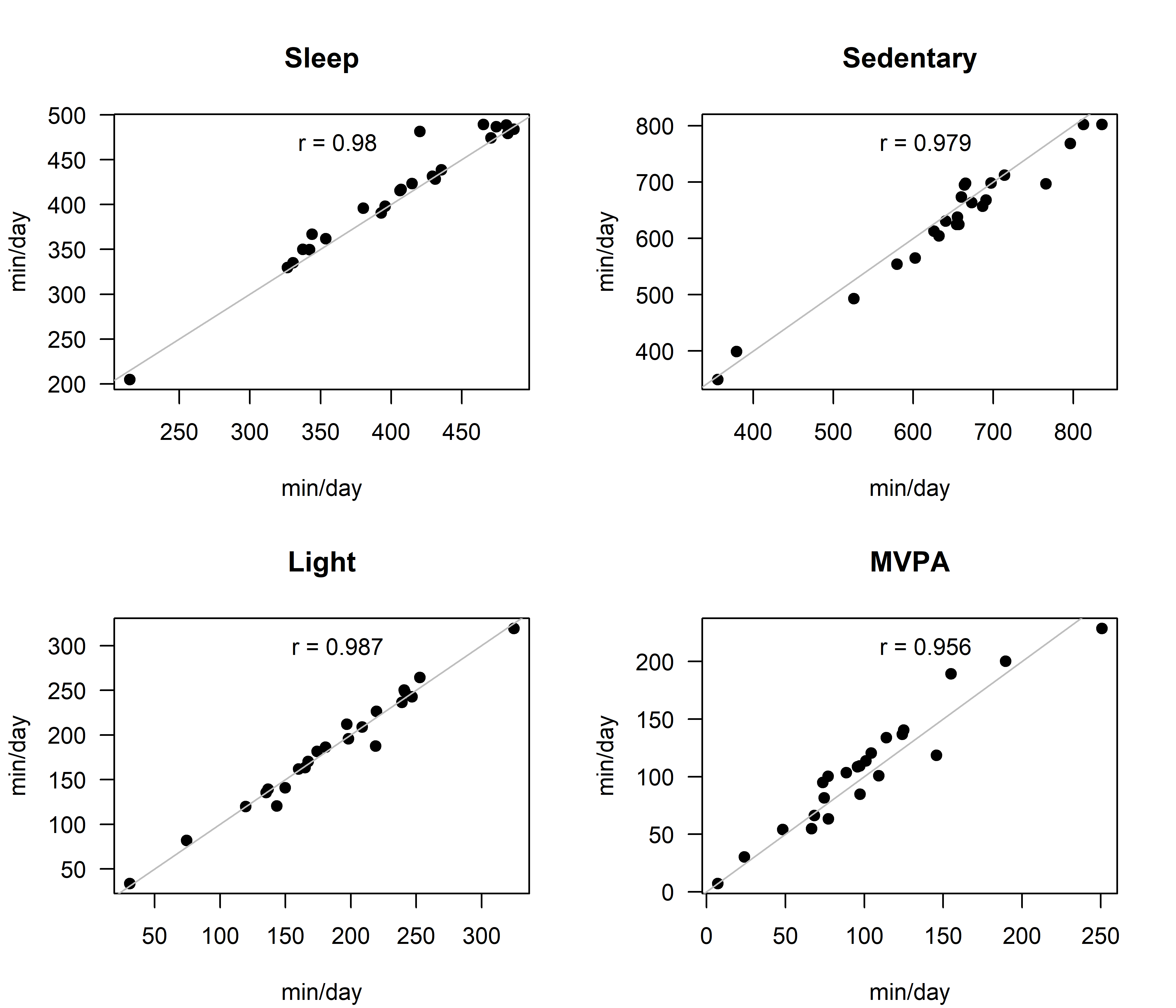
**

**Figure S2.** Scatter plots for the correlation between **the Movisens and the GENEActiv** in the definition of sleep, sedentary time, light PA, and MVPA. The grey line represents the perfect correlation (i.e., r = 1).

MVPA: moderate-to-vigorous physical activity; PA: physical activity.

**
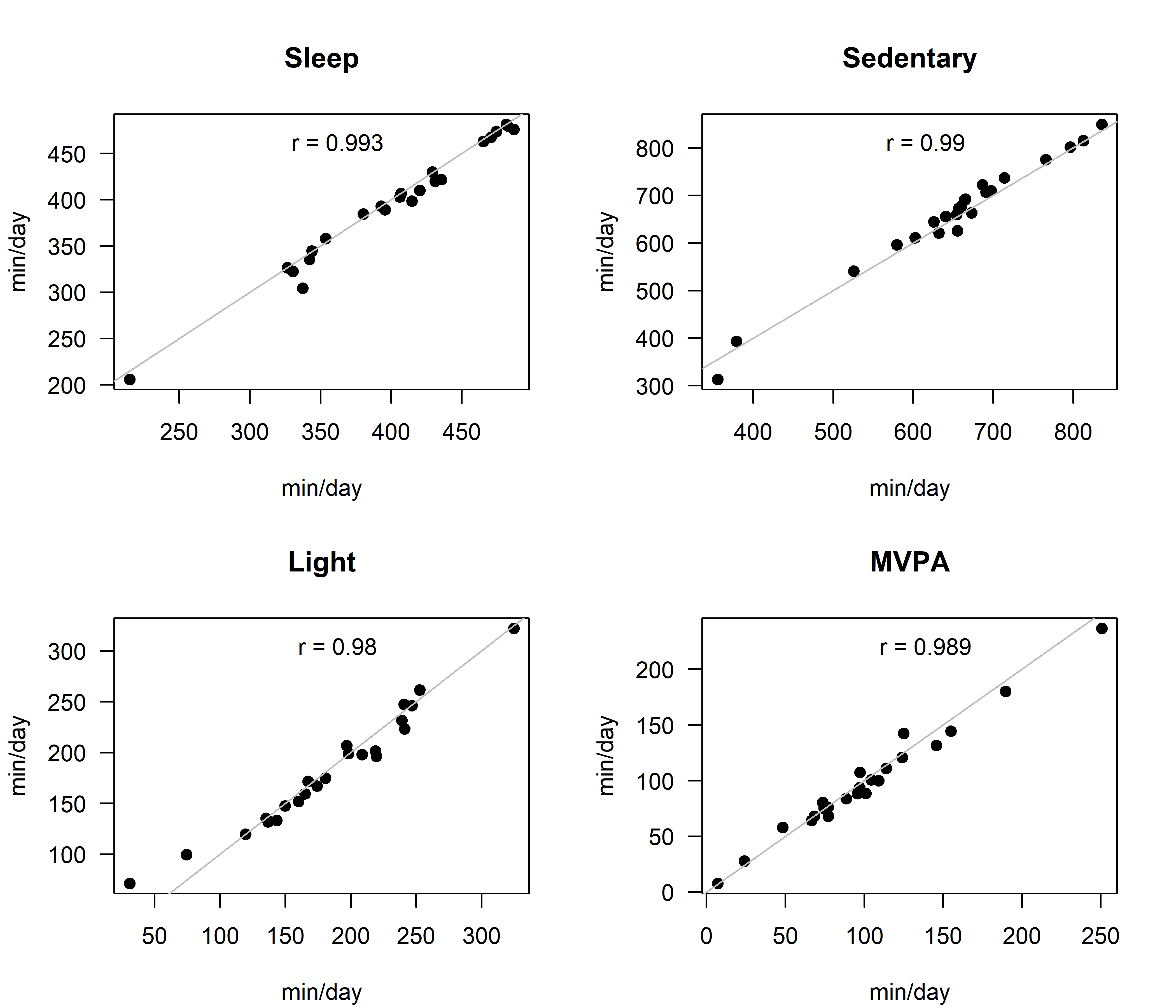
**

**Figure S3.** Scatter plots for the correlation between **the Movisens and the Axivity** in the definition of sleep, sedentary time, light PA, and MVPA. The grey line represents the perfect correlation (i.e., r = 1).

MVPA: moderate-to-vigorous physical activity; PA: physical activity.

**
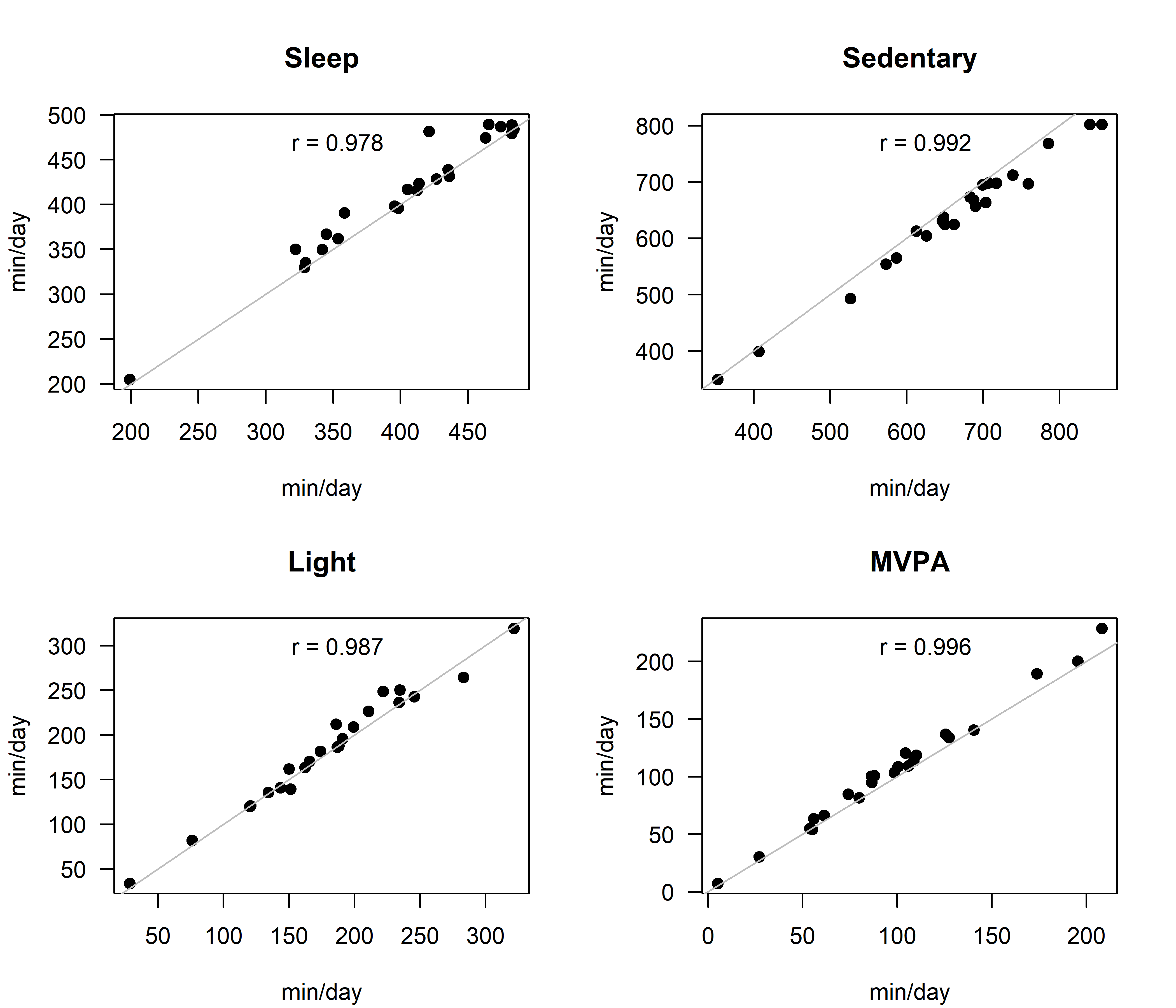
**

**Figure S4.** Scatter plots for the correlation between **the ActiGraph and the GENEActiv** in the definition of sleep, sedentary time, light PA, and MVPA. The grey line represents the perfect correlation (i.e., r = 1).

MVPA: moderate-to-vigorous physical activity; PA: physical activity.

**
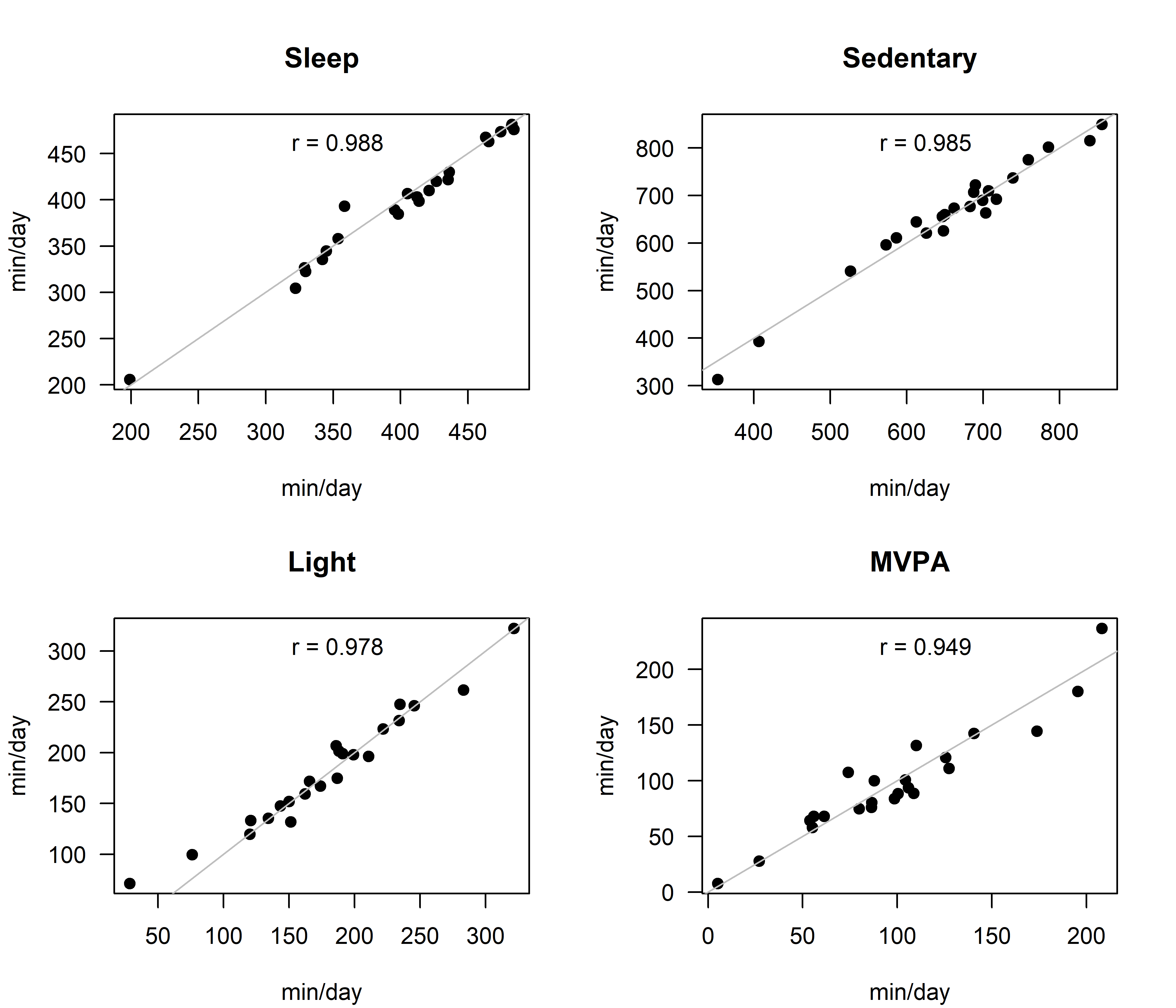
**

**Figure S5.** Scatter plots for the correlation between **the ActiGraph and the Axivity** in the definition of sleep, sedentary time, light PA, and MVPA. The grey line represents the perfect correlation (i.e., r = 1).

MVPA: moderate-to-vigorous physical activity; PA: physical activity.

**
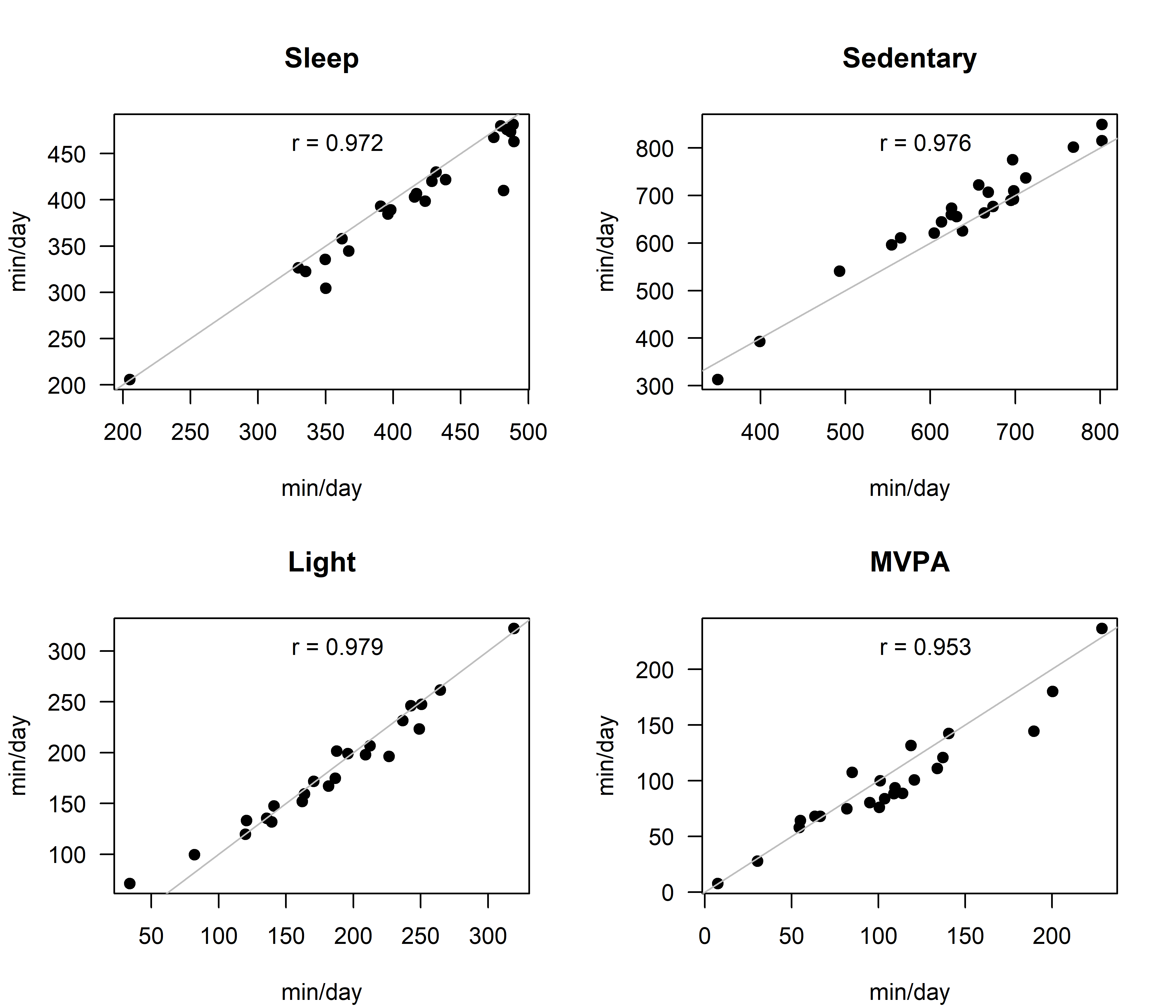
**

**Figure S6.** Scatter plots for the correlation between **the GENEActiv and the Axivity** in the definition of sleep, sedentary time, light PA, and MVPA. The grey line represents the perfect correlation (i.e., r = 1).

MVPA: moderate-to-vigorous physical activity; PA: physical activity.

**
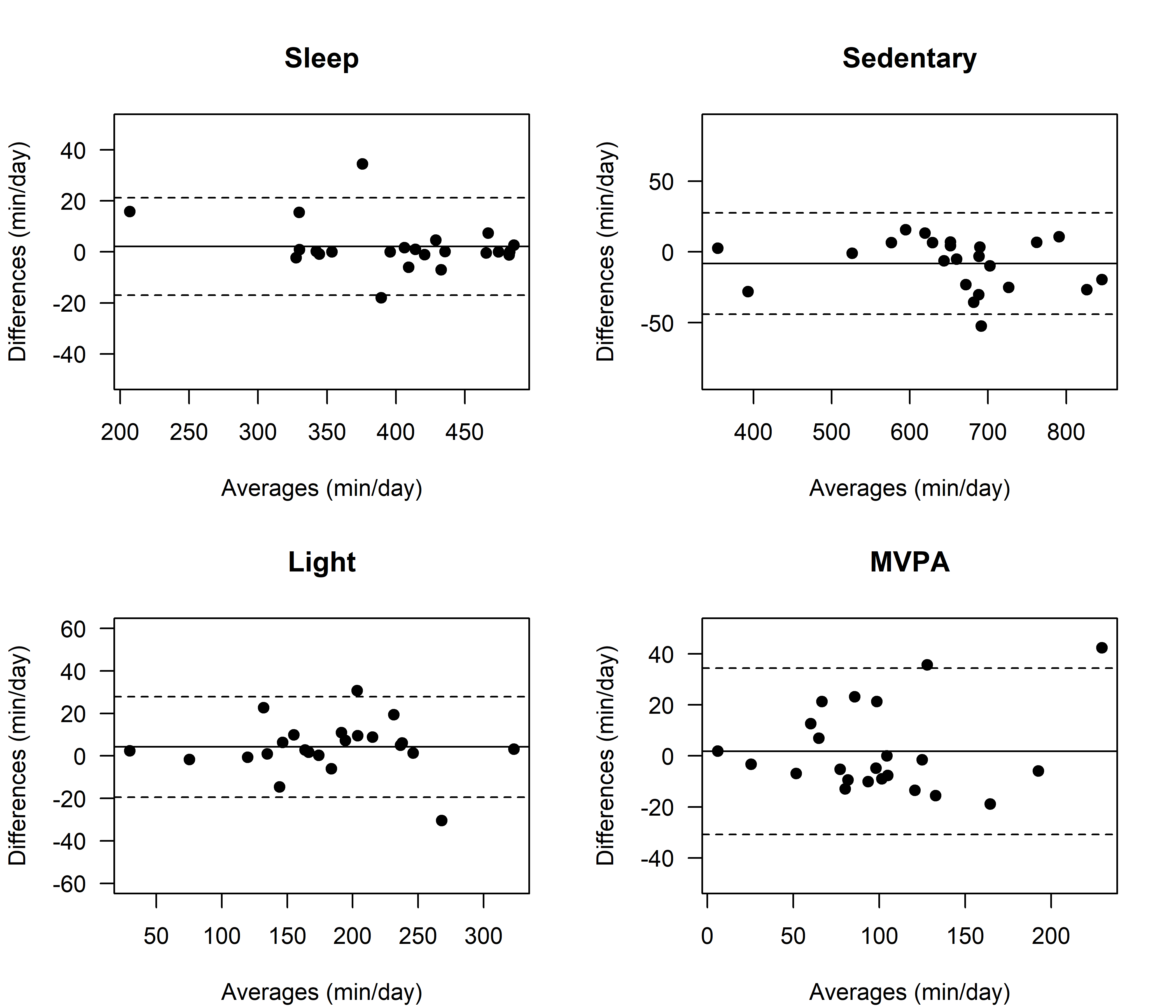
**

**Figure S7.** Bland-Altman plots for the agreement between **the Movisens and the ActiGraph** in the definition of sleep, sedentary time, light PA, and MVPA. The solid line represents the mean bias, and the dashed lines represent the 95% limits of agreement.

MVPA: moderate-to-vigorous physical activity; PA: physical activity.

**
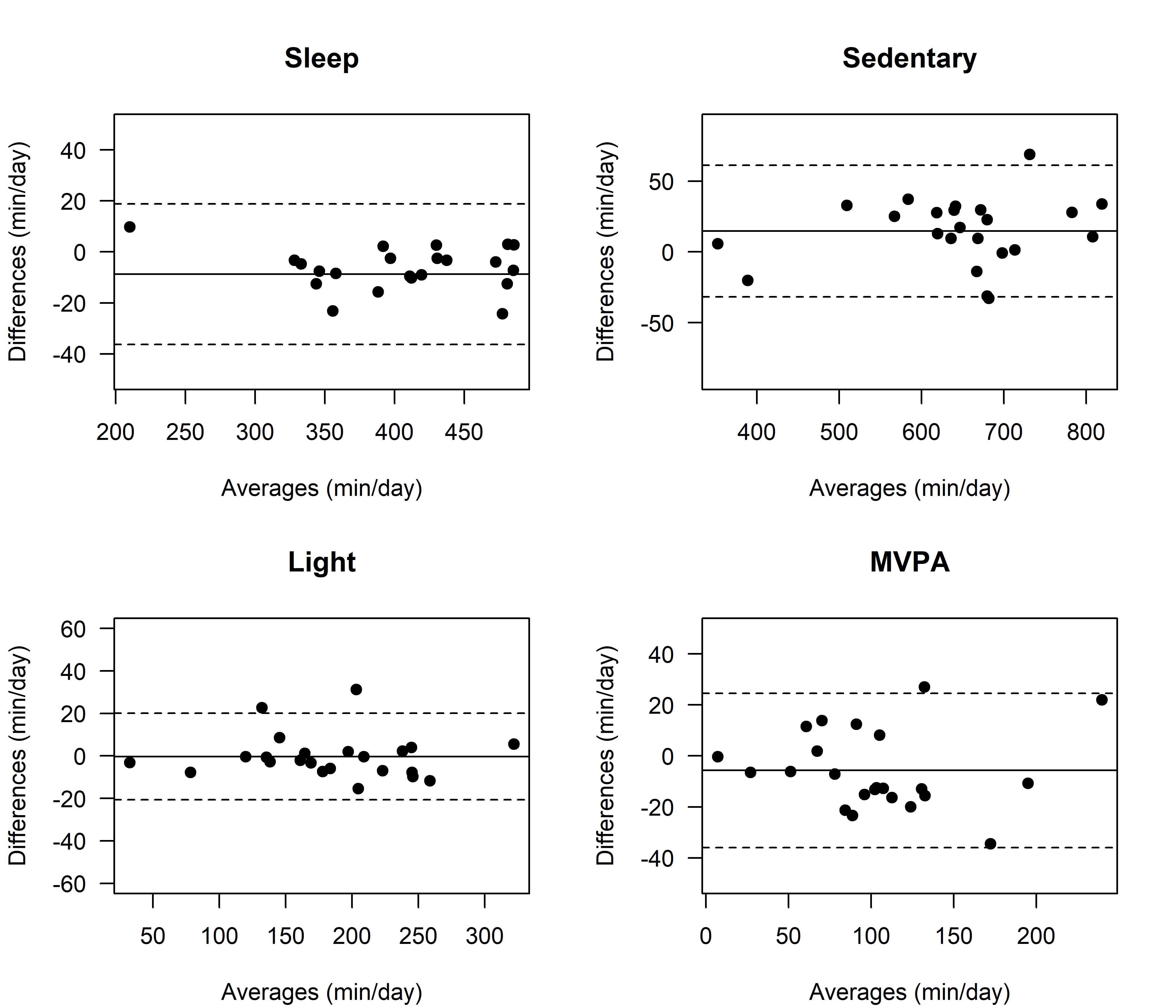
**

**Figure S8.** Bland-Altman plots for the agreement between **the Movisens and the GENEActiv** in the definition of sleep, sedentary time, light PA, and MVPA. The solid line represents the mean bias, and the dashed lines represent the 95% limits of agreement.

MVPA: moderate-to-vigorous physical activity; PA: physical activity.

**
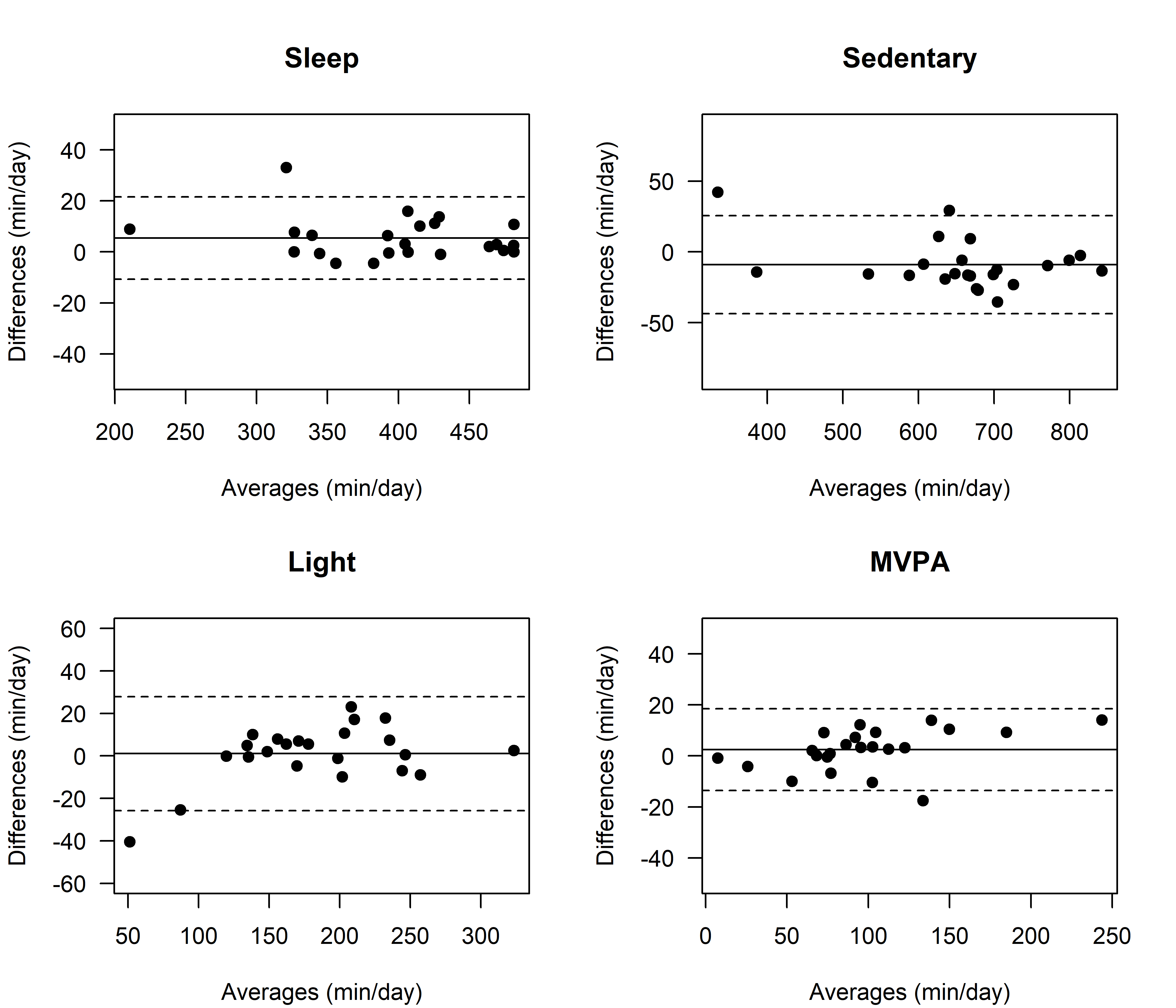
**

**Figure S9.** Bland-Altman plots for the agreement between **the Movisens and the Axivity** in the definition of sleep, sedentary time, light PA, and MVPA. The solid line represents the mean bias, and the dashed lines represent the 95% limits of agreement.

MVPA: moderate-to-vigorous physical activity; PA: physical activity.

**
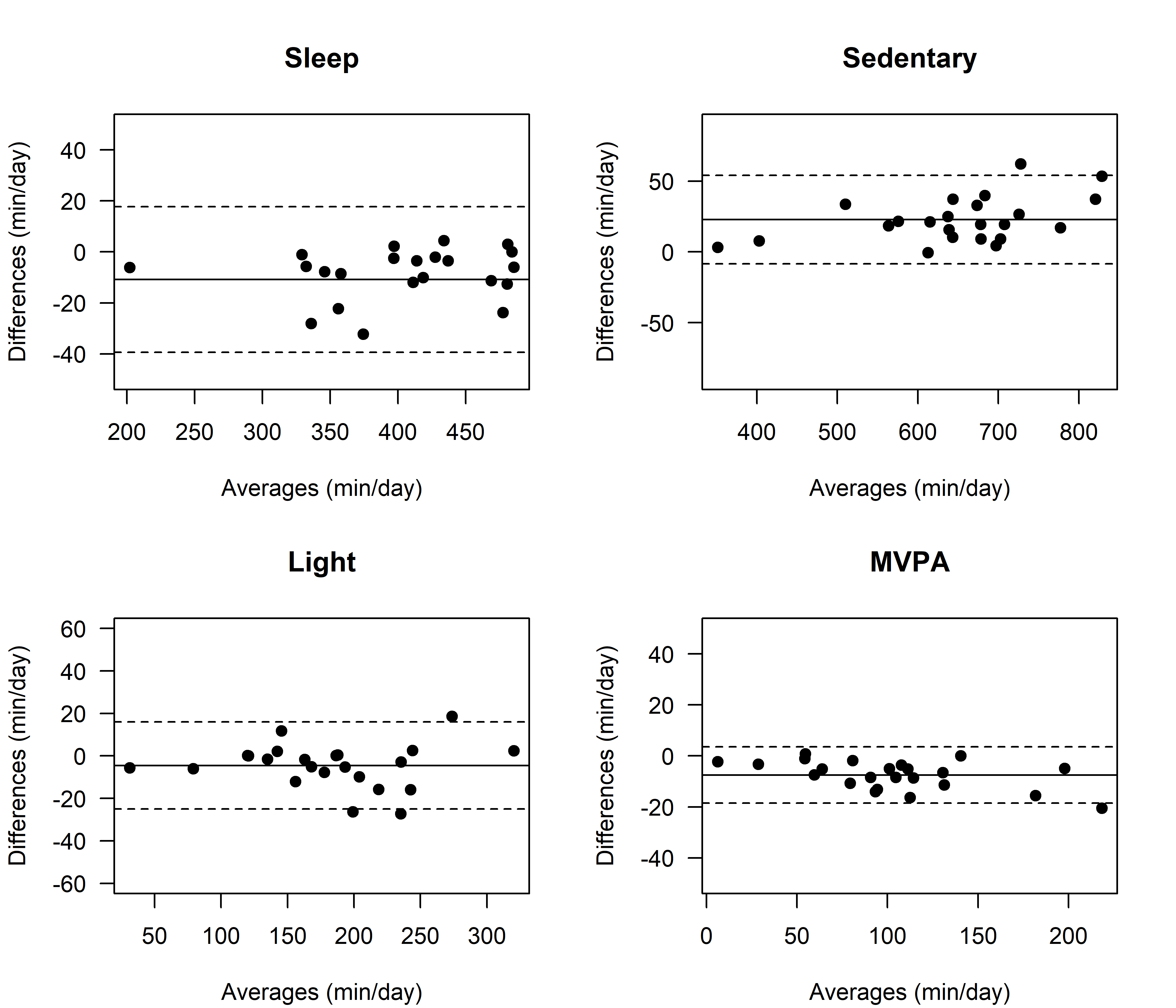
**

**Figure S10.** Bland-Altman plots for the agreement between **the ActiGraph and the GENEActiv** in the definition of sleep, sedentary time, light PA, and MVPA. The solid line represents the mean bias, and the dashed lines represent the 95% limits of agreement.

MVPA: moderate-to-vigorous physical activity; PA: physical activity.

**
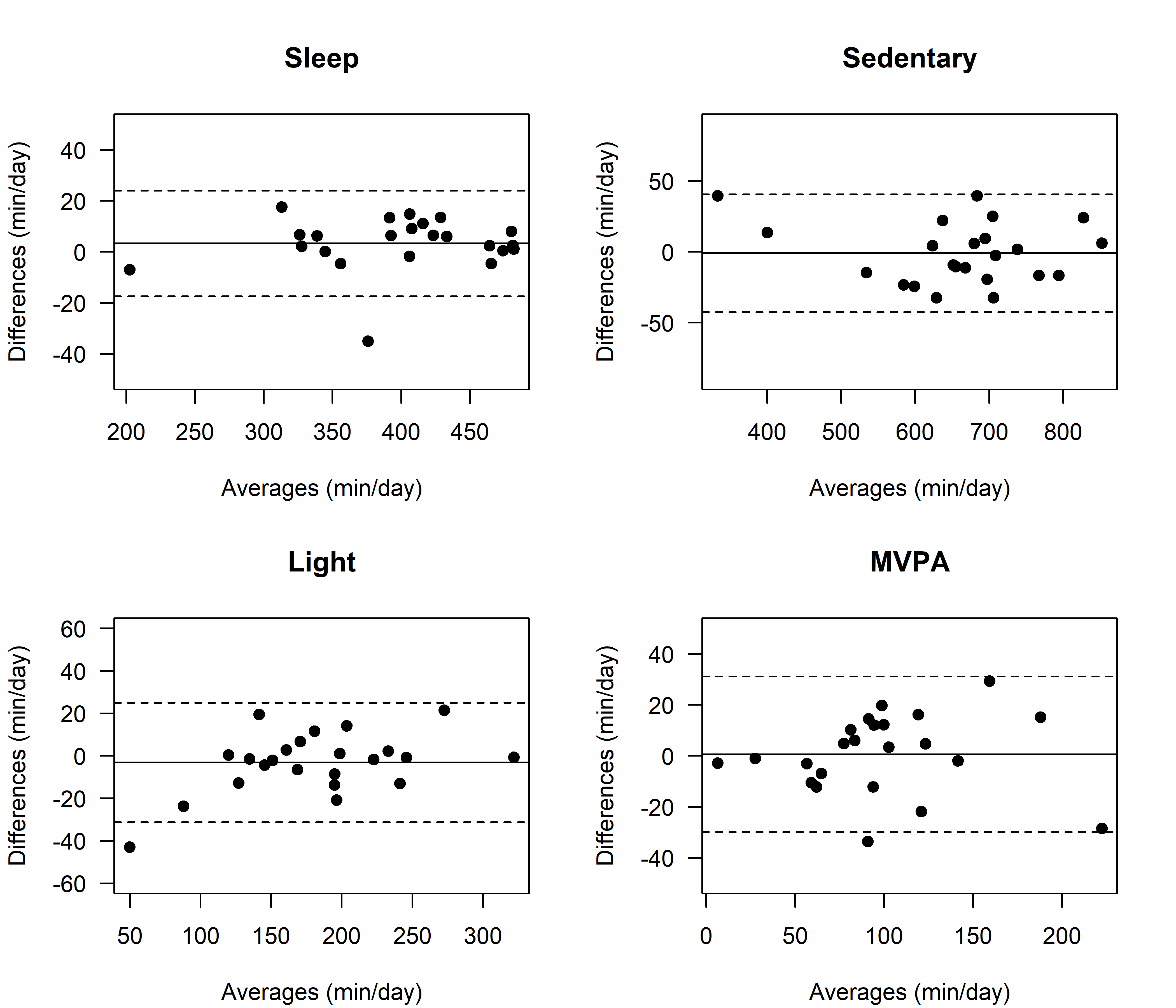
**

**Figure S11.** Bland-Altman plots for the agreement between **the ActiGraph and the Axivity** in the definition of sleep, sedentary time, light PA, and MVPA. The solid line represents the mean bias, and the dashed lines represent the 95% limits of agreement.

MVPA: moderate-to-vigorous physical activity; PA: physical activity.

**
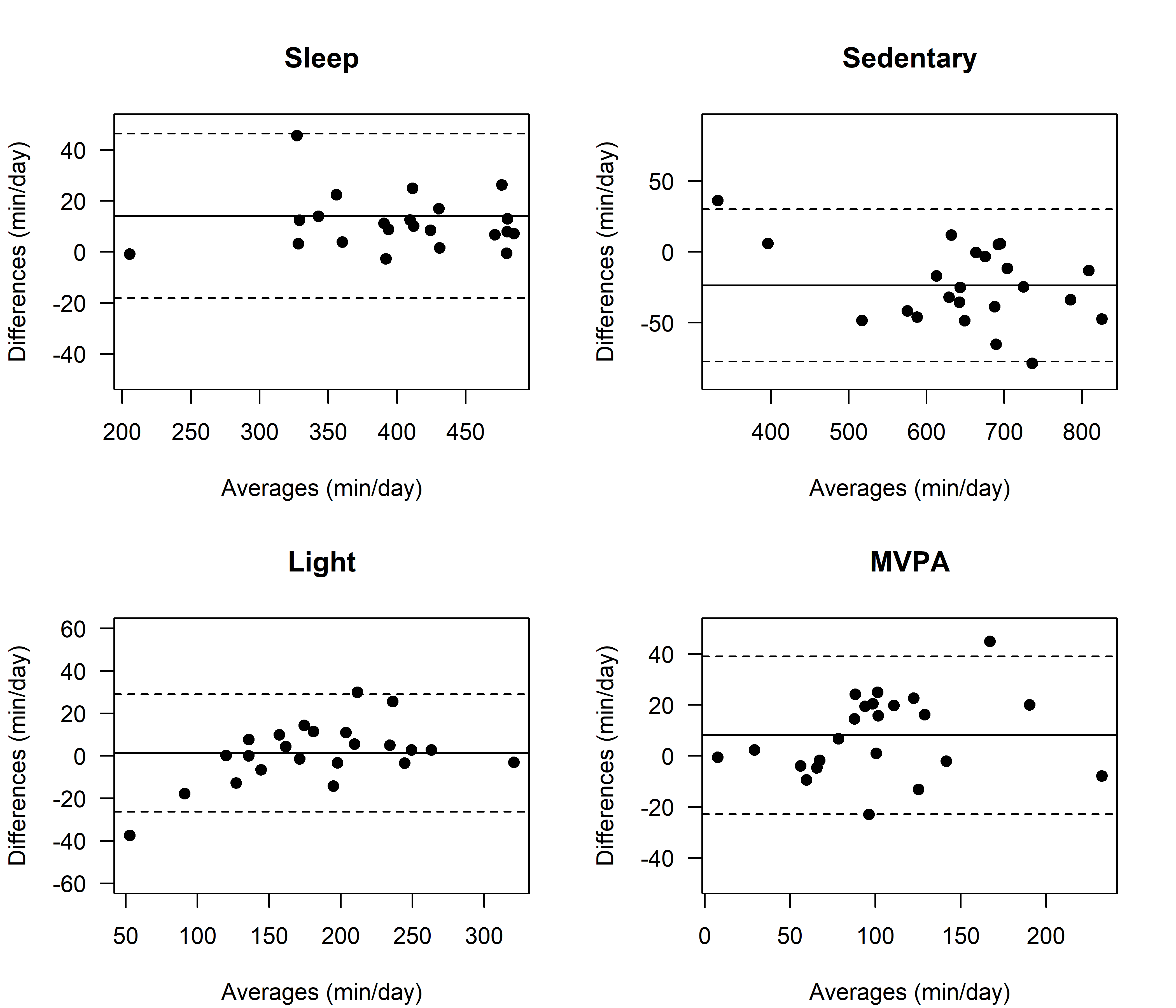
**

**Figure S12.** Bland-Altman plots for the agreement between **the GENEActiv and the Axivity** in the definition of sleep, sedentary time, light PA, and MVPA. The solid line represents the mean bias, and the dashed lines represent the 95% limits of agreement.

MVPA: moderate-to-vigorous physical activity; PA: physical activity.
